# A dynamic Bayesian network approach to protein secondary structure prediction

**DOI:** 10.1186/1471-2105-9-49

**Published:** 2008-01-25

**Authors:** Xin-Qiu Yao, Huaiqiu Zhu, Zhen-Su She

**Affiliations:** 1 State Key Laboratory for Turbulence and Complex Systems and Department of Biomedical Engineering, Peking University, Beijing, 100871, China; 2 Center for Theoretical Biology, Peking University, Beijing, 100871, China; 3 Department of Mathematics, University of California, Los Angeles, Los Angeles, CA 90095, USA

## Abstract

**Background:**

Protein secondary structure prediction method based on probabilistic models such as hidden Markov model (HMM) appeals to many because it provides meaningful information relevant to sequence-structure relationship. However, at present, the prediction accuracy of pure HMM-type methods is much lower than that of machine learning-based methods such as neural networks (NN) or support vector machines (SVM).

**Results:**

In this paper, we report a new method of probabilistic nature for protein secondary structure prediction, based on dynamic Bayesian networks (DBN). The new method models the PSI-BLAST profile of a protein sequence using a multivariate Gaussian distribution, and simultaneously takes into account the dependency between the profile and secondary structure and the dependency between profiles of neighboring residues. In addition, a segment length distribution is introduced for each secondary structure state. Tests show that the DBN method has made a significant improvement in the accuracy compared to other pure HMM-type methods. Further improvement is achieved by combining the DBN with an NN, a method called DBNN, which shows better *Q*_3 _accuracy than many popular methods and is competitive to the current state-of-the-arts. The most interesting feature of DBN/DBNN is that a significant improvement in the prediction accuracy is achieved when combined with other methods by a simple consensus.

**Conclusion:**

The DBN method using a Gaussian distribution for the PSI-BLAST profile and a high-ordered dependency between profiles of neighboring residues produces significantly better prediction accuracy than other HMM-type probabilistic methods. Owing to their different nature, the DBN and NN combine to form a more accurate method DBNN. Future improvement may be achieved by combining DBNN with a method of SVM type.

## Background

Over past decades, the prediction accuracy of protein secondary structure has gained some improvements, largely due to the successful application of machine learning tools such as neural network (NN) and support vector machine (SVM). Qian and Sejnowski designed one of the earliest NN methods [1]. Rost and Sander introduced the alignment profile with multiple sequence alignment into the prediction. Their method, named as PHD, performed much better than previous ones, because of the use of alignment profile as the network's input [2]. Jones made an important improvement by pioneering the use of position-specific scoring matrices (PSSM) to generate the so-called PSI-BLAST profile and developed the method called PSIPRED [3]. Recently, new advances have been made in developing NN-based prediction methods [4-7]. Similarly, SVM-based methods were developed for protein secondary structure prediction, first taking the alignment profile as inputs and then being improved to use the PSI-BLAST profile [8-12]. Generally speaking, the *Q*_3 _of a modern NN or SVM-based method can reach over 76%.

In contrast to NN and SVM, probabilistic methods for protein secondary structure prediction such as those based on hidden Markov model (HMM) have had very limited accuracy [13-18]. Most of them were designed for single sequence prediction with prediction accuracy generally less than 70%. Recently, two profile-based HMM methods were proposed, which take either the alignment profile or PSI-BLAST profile as inputs [16,18]. Both of the methods treat the profile as production from a multinomial distribution with 20 possible outcomes (20 amino acids), and thus lose the information about the correlation between entries of the profile. As a result, the prediction accuracy of the two methods, which is around 72%, is still much lower than the common level of NN or SVM-based methods. It is notable that there is a special HMM-type method, SAM-T04 [19], which has shown comparable accuracy to NN and SVM-based methods. However, with using a neural network for the sequence-to-structure prediction while building the HMM only at the secondary structure level [19,20], SAM-T04 should not be regarded as a pure HMM-type method.

It would be interesting to break this apparent asymmetry in accuracy between machine learning-based methods and probabilistic model-based methods. The probabilistic model is of somewhat different nature from machine learning tools, and provides a complement to the latter. Thus, combining the two kinds of model is likely to produce a consensus prediction that has better accuracy than the prediction of individual program [21]. In addition, the probabilistic model outputs a set of knowledge about the property of secondary structure in an explicit way, including specific correlation structure between neighboring residues, while such information is implicit in NN or SVM. Hence, the development of an appropriate probabilistic model is interesting for understanding the mechanism by which sequence determines structure.

In this paper we introduce a new probabilistic model, dynamic Bayesian network (DBN), for protein secondary structure prediction. DBN represents a directed graphical model of a stochastic process, often regarded as a generalized HMM capable of describing correlation structure in a more flexible way [22]. A novel feature of our method is the introduction of a multivariate Gaussian distribution for the profile of each residue, which takes into account the correlation between entries of the PSSM. In addition, our method considers a high-ordered dependency between profiles of neighboring residues and introduces a segment length distribution for each secondary structure state. Testing results show that the DBN method has made a significant improvement in accuracy over previous pure HMM-type methods. Further improvement is achieved by combining the DBN with an NN, a method named DBNN, which has achieved better *Q*_3 _accuracy than many other popular methods and is competitive to the current state-of-the-arts. The most interesting feature of DBN/DBNN is that a significant improvement in the prediction accuracy is achieved when combined with other methods by a simple consensus.

## Results and Discussion

### Training and testing datasets

Three public datasets are employed for training and testing, i.e. CB513 [21], EVA [23] common set, and a large dataset containing 3,223 chains (denoted by EVAtrain) constructed by G. Karypis [12]. The first dataset contains 513 protein sequences with guaranteed non-redundancy via a strict criterion (z-score ≥ 5) for the sequence similarity; this dataset is used independently from two other datasets. The second is obtained from EVA server, where several secondary structure prediction servers are evaluated with sequences deposited in PDB [24]. In particular, a set labeled as "common set 6" (denoted by EVAc6) is selected, which contains 212 protein chains and has been used to test several popular prediction methods [25]. The third dataset, EVAtrain, is used in conjunction with EVAc6, with the former for training and the latter for testing. EVAtrain has been guaranteed to have less than 25% sequence identities to EVAc6.

Furthermore, we have built a fourth dataset based on the known tertiary structural similarity from the SCOP [26] database (release 1.69), to evaluate the performance of our methods when dealing with proteins of remote evolutionary relation. One protein domain for each superfamily of the four classes (all *α*, all *β*, *α *and *β*, *α*/*β*) is selected. The domains of multi-segment, of NMR structure, and of low X-ray resolution (> 2.5Å) are removed. Also, too short (< 30 residues) or too long (> 500 residues) sequences are removed. The final dataset contains 576 protein sequences and is referred to as SD576.

For all the datasets described above, the secondary structure is assigned by DSSP program [27], and the eight-state secondary structure is converted to three, according to the rule: H, G, and I to H (helix); E and B to E (sheet); all others to C (coil).

### Window sizes

The window sizes, denoted by *L*_*AA *_and *L*_*SS *_for profile and secondary structure respectively, describe the range of dependency of current site on its neighbors. The correlation between the *Q*_3 _accuracy of DBN and window sizes is studied via a set of seven-fold cross-validation tests of DBN_sigmoid _(see Methods) on SD576 using different window sizes. Due to the limitation in the computational resources, the upper bounds of *L*_*AA *_and *L*_*SS *_are set to be 5 and 4, respectively.

**Figure 1:**
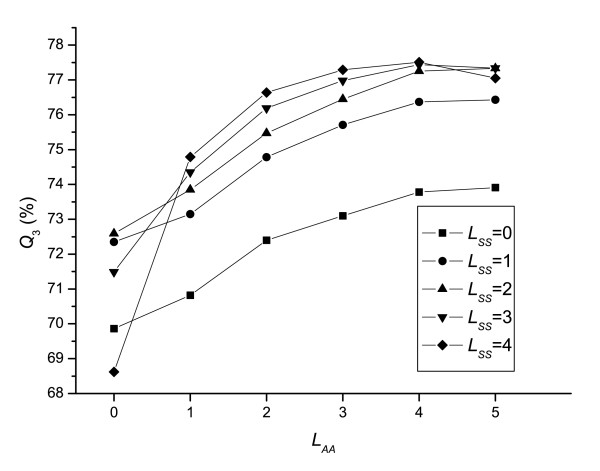
**The influence of window sizes on the *Q*_3 _of DBN**. L_*AA *_and L_*SS *_are window sizes for profile and secondary structure, respectively. The results are obtained by testing DBN_sigmoid _on the SD576 dataset.

**Figure 2:**
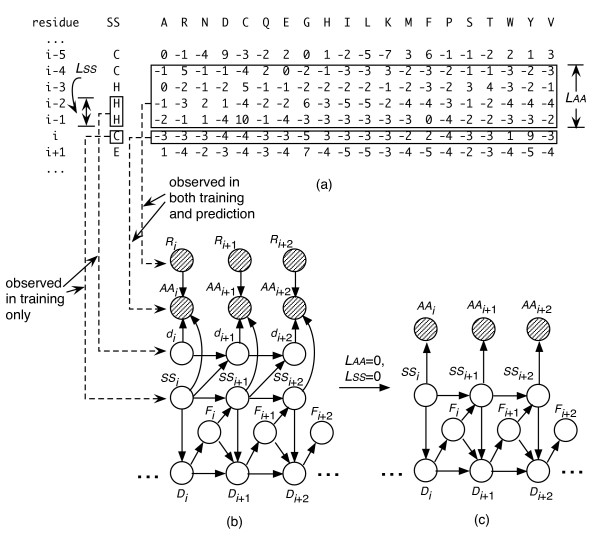
**Illustration of the DBN model**. (a) An example of PSSM, where rows represent residue sites and columns represent amino acids. The "SS" column contains the secondary structure of each site, classified as H (helix), E (sheet), and C (coil). (b) A graphical representation of the DBN. The shadow nodes represent observable random variables, while clear nodes represent hidden (in prediction) variables. The arcs with arrows represent dependency between nodes. The contents of the nodes *R*_*i*_, *AA*_*i*_, *d*_*i*_, and *SS*_*i *_are derived as illustrated by the connections of dashed lines, where the subscript indicates the residue site. More detailed description of *R*_*i*_, *AA*_*i*_, *d*_*i*_, *SS*_*i*_, *D*_*i*_, and *F*_*i *_can be found in the text. *L*_*AA *_and *L*_*SS *_are windows sizes for profile and secondary structure, respectively (in this example, *L*_*AA *_= 4 and *L*_*SS *_= 2). (c) Is a reduced version of (b) with *L*_*AA *_= 0 and *L*_*SS *_= 0.

As shown in Fig. 1, *Q*_3 _is improved significantly when *L*_*SS *_> 0, and saturated when *L*_*SS *_> 1, which indicates that there is strong short-range dependency between the profile of a residue and the secondary structure states of its neighbors. A similar phenomenon occurs for profiles' dependency of neighboring sites. Note that the model with either *L*_*AA *_= 0 or *L*_*SS *_= 0 is a special case of DBN, in which the distribution of the profile of each residue is independent from neighboring profiles or neighboring secondary structure states, respectively. As a result, its topology is different from that of a full-DBN version (*L*_*AA *_> 0 and *L*_*SS *_> 0) due to the removal of *R*_*i *_or *d*_*i *_nodes (see Fig. 2(c)).

Our results are in partial agreement with the conclusions of Crooks and Brenner, who claimed that each amino acid was dependent on the neighboring secondary structure states but was essentially independent from neighboring amino acids [16]. We argue, however, that the PSI-BLAST profile has quite different correlation structure from a single amino acid sequence, from which Crooks *et al*. derived their conclusions. In fact, the dependency between neighboring profiles are significant and helpful for improving the prediction accuracy.

Fig. 1 also shows that the most accurate model occurs when using the set (*L*_*AA *_= 4, *L*_*SS *_= 4), for which *Q*_3 _reaches about 77.5%. However, test shows that this model is very time-consuming. We choose a more economical set (*L*_*AA *_= 4, *L*_*SS *_= 3) which offers a similar *Q*_3 _(see Fig. 1) with a big saving in computational cost, for all the DBN models used in current study.

### The accuracy improvements through combinations

All the basic DBN- and NN-based models described in Methods are tested on the SD576 dataset, and the results shown in Table 1 report the performance of these models, as well as of their combinations. Specifically, both DBN_linear _(combination of DBN_linear+NC _and DBN_linear+CN_) and DBN_sigmoid _(combination of DBN_sigmoid+NC _and DBN_sigmoid+CN_) have significantly improved the performance in all the measures, indicating that the two directions of the sequence (i.e. from N-terminus to C-terminus and reverse) contain complementary information. In addition, the combination of the two different PSSM-transformation strategies (i.e. the combination of DBN_linear _and DBN_sigmoid _to produce DBN_final_) also contributes to the accuracy improvement, increasing *Q*_3 _and *SOV *by 0.8% and 0.9%, respectively, for DBN-based models. Note that for NN-based models, the accuracy improvement by combination is much less evident, indicating that NN is not sensitive to PSSM-transformation strategies.

**Table 1:** Performance of basic DBN and NN models and their combinations tested on SD576.

Model	*Q*_3 _(%)	*SOV *(%)	*C*_H_	*C*_E_	*C*_C_
DBN_linear+NC_	75.1	74.0	0.69	0.60	0.55
DBN_linear+CN_	74.6	73.3	0.68	0.61	0.53
DBN_linear_	77.0	75.8	0.72	0.64	0.58
DBN_sigmoid+NC_	75.8	74.5	0.72	0.60	0.56
DBN_sigmoid+CN_	74.6	73.3	0.69	0.61	0.54
DBN_sigmoid_	77.4	75.9	0.74	0.64	0.59
DBN_final_	78.2	76.8	0.74	0.65	0.60
NN_linear_	77.6	73.2	0.72	0.64	0.60
NN_sigmoid_	77.1	71.0	0.72	0.63	0.59
NN_final_	77.8	73.3	0.73	0.64	0.60
DBNN	80.0	78.1	0.77	0.68	0.63

All the eleven models listed in the table are described in Methods. The average results of seven-fold cross-validation are shown.

Table 1 shows that DBN_final _has improved by 3.5% over NN_final _in *SOV*. It can be understood, because DBN-based models explicitly incorporate the segment length distributions while NN-based models miss such information.

Finally, the combination of all the basic DBN- and NN-based models, which produces the resultant DBNN, has achieved further improvement in the accuracy, increasing *Q*_3 _and *SOV *by 1.8% and 1.3%, respectively, compared to DBN_final _(see Table 1). This implies that the two types of models are indeed complementary.

### Secondary structure segment length distributions

**Figure 3:**
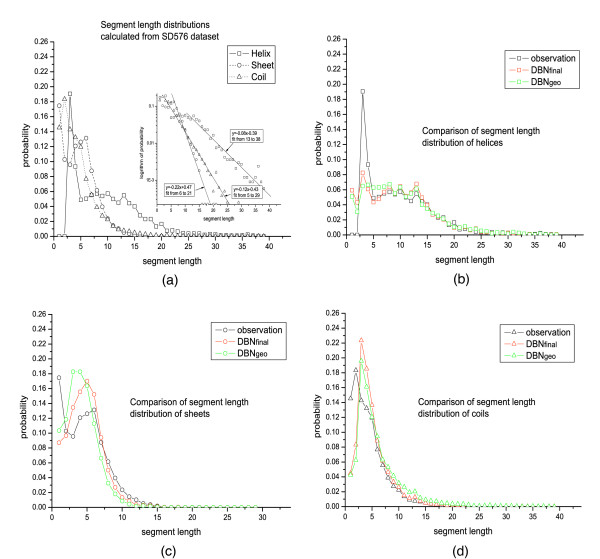
**Segment length distributions of helices, sheets, and coils**. (a) The observed distributions calculated directly from SD576 dataset. Inset is lin-log plots of the distributions, where the lines show fitting exponential tails for the three types of secondary structure segments. (b) The comparison between the distribution of helices observed in the dataset and those predicted by DBN_final _and DBN_geo_. (c) The comparison of distributions between observation and prediction of sheets. (d) The comparison of distributions between observation and prediction of coils.

To study the significance of the secondary structure segment length distributions introduced in DBN models, we define a degenerate DBN (denoted by DBN_geo_), which has the same structure to DBN_final _except *D*_*max *_= 1 [see Eq. (10)]. As described in Methods, *D*_*max *_= 1 implies a geometric distribution for the segment lengths. The segment length distributions of the predicted secondary structure by both DBN_final _and DBN_geo _are calculated and compared to the true distributions observed in the SD576 dataset, as shown in Fig. 3(b)–(d). In particular, Fig. 3(b) shows that, for helices, the segments of one and two residues are over-predicted, while those of three residues are under-predicted, by both DBN_final _and DBN_geo_. But longer segments are all predicted correctly by both models. Generally speaking, DBN_final _has better performance than DBN_geo_: the prediction of DBN_final _for segments of 3 and 5–7 residues is much better than that of DBN_geo_.

Fig. 3(c) and 3(d) show the segment length distributions for sheets and coils, respectively. Both DBN_final _and DBN_geo _have missed a rich population of one residue, and over-predicted segments of 3–5 residues, for sheets. DBN_geo _has predicted a spurious peak for segments of 3 and 4 residues, which is absent in the true distribution. On the contrary, DBN_final _gives a distribution closer to the observation, in which the peak is located at segments of about 5 residues. Fig. 3(d) shows that DBN_final _and DBN_geo _have very similar performance for coils: both under-predict the segments of 1 and 2 residues and over-predict those of 3 and 4 residues. However, DBN_final _predicts a much better distribution for long coils (over 8 residues) than DBN_geo_.

It is interesting to study whether we can modify the *a priori *segment length distribution, *g*_*α*_(*n*) in Eq. (10), to get a predicted (posterior) distribution closer to the observation shown in Fig. 3(a). A calculation is made by using a modified version of DBN_final_, denoted by DBN_mod_, which is constructed as following: take the *a priori *segment length distribution directly from the training set, then run the prediction and calculate the posterior distribution, and finally modify the *a priori *distribution according to the following equation:

(1) $$g_{\alpha }^{new}(n)=\max\{g_{\alpha }^{old}(n)+1.5[g_{\alpha }^{obs}(n)-g_{\alpha }^{pre}(n)],\;0\},$$

where *g*_*α*_^*old*^(*n*) is the *a priori *segment length distribution before the modification, *g*_*α*_^*pre*^(*n*) is the predicted distribution, *g*_*α*_^*obs*^(*n*) is the observed distribution, *α *= H, E, or C, and *n *= 1, 2, ... *D*_*max*_. The quantity *g*_*α*_^*new*^(*n*) is then normalized to form the new *a priori *segment length distribution. The Eq. (1) enhances the population of deficient segments and reduces that of over-represented ones, in a linear fashion. All the three models, DBN_final_, DBN_geo_, and DBN_mod_, are tested on SD576, and the performance on segment length distributions prediction is measured by "relative entropies", defined by

(2) $$H_{\alpha }=\sum_{n=1}^{D_{\max}}g_{\alpha }^{obs}(n)\cdot \log_{2}\frac{g_{\alpha }^{obs}(n)}{g_{\alpha }^{pre}(n)},$$

where *g*_*α*_^*obs*^(*n*), *g*_*α*_^*pre*^(*n*), and *D*_*max *_have the same definitions as above, and *α *= H, E, or C.

The results presented in Table 2 show that DBN_geo _has much higher relative entropies indicating a strong deviation of the predicted distributions from the observation, than other two models. Note that *Q*_3 _and *SOV *of DBN_geo _are also much lower than that of DBN_final _(Table 2), implying that the segment length distributions do have an effect on the prediction accuracy. On the other hand, DBN_mod _shows the lowest relative entropies for all the three secondary structure states with almost the same *Q*_3 _and *SOV *to DBN_final _(see Table 2), which indicates that Eq. (1) has effectively improved the prediction of segment length distributions.

**Table 2:** Performance of DBN_geo_, DBN_final_, and DBN_mod _tested on SD576.

Model	*Q*_3 _(%)	*SOV *(%)	Relative entropy (bit)			
			
			Helix	Sheet	Coil	Average
DBN_geo_	76.7	74.3	0.247	0.170	0.290	0.236
DBN_final_	78.2	76.8	0.236	0.096	0.210	0.181
DBN_mod_	78.2	76.3	0.214	0.038	0.110	0.121

The seven-fold cross-validation test results on three models with different segment length distributions are explained in the text. The performance is measured by *Q*_3_, *SOV*, and the relative entropies between the observed segment length distributions from SD576 and the model's predictions [Eq. (2)]. Clearly, DBN_final _and DBN_mod _have visible improvement over DBN_geo_.

### Comparison between DBN and leading HMM-type methods

The DBN method (DBN_final_) developed in this work is also evaluated on the widely used CB513 dataset, and its performance is compared to two recently published HMM-type methods, denoted by HMMCrooks [16] and HMMChu [18], respectively, both of which have also been tested on the same or a similar dataset. In comparison, we have calculated the significant-difference margin (denoted by ErrSig) for each score, which is defined as the standard deviation divided by the square root of the number of proteins and was used by others [12]. The results presented in Table 3 show that DBN_final _has made improvements for all measures compared to the two methods mentioned above. Specifically, DBN_final _improves *Q*_3 _by 3.5% over HMMCrooks and 4.1% over HMMChu, and improves *SOV *by 4.4% over HMMChu. Since the ErrSig for *Q*_3 _and *SOV *are 0.41 and 0.63, respectively, the improvements are judged to be significant. Matthews' coefficients [28] shown in Table 3 indicate that DBN_final _is particularly good at the prediction of helices and sheets, compared to above two methods.

**Table 3:** Comparative performance of DBN_final _and DBN_diag _against leading HMM-type methods tested on CB513.

Method	*Q*_3 _(%)	*SOV *(%)	*C*_H_	*C*_E_	*C*_C_
HMMCrooks	72.8	--	--	--	--
HMMChu	72.2	68.3	0.61	0.52	0.51
DBN_diag_/ErrSig	72.5/0.42	65.9/0.63	0.66/0.01	0.55/0.01	0.51/0.01
DBN_final_/ErrSig	76.3/0.41	72.7/0.63	0.71/0.01	0.61/0.01	0.57/0.01

DBN_final _and DBN_diag _are methods developed in this work and their descriptions can be found in the text. Entries marked with "--" mean that the data could not be obtained from the literature. HMMChu has been trained and tested on the CB480 dataset (a reduced version of CB513), while all other methods have been trained and tested on the CB513 dataset. The average results of seven-fold cross-validation are shown.

The improvements made by DBN_final _are believed mainly due to the use of a conditional linear Gaussian distribution to model the PSI-BLAST profile of each residue, in which the correlation between the 20 entries in the profile is considered (see Methods). In contrast, both HMMCrooks and HMMChu employ a multinomial distribution to model the profile, which lacks the above correlation information [16,18]. The supporting experiment of our conjecture consists in constructing a degenerate DBN model (denoted by DBN_diag_) that has the similar architecture to DBN_final _but only has a diagonal covariance matrix for the distribution of *AA*_*i *_[Eq. (7)], so that the correlation between entries of the profile is ignored. We have tested this model on the CB513 dataset, and the results (Table 3) show that the *Q*_3 _of DBN_diag _drops down to 72.5%, similar to those of HMMCrooks and HMMChu, which highlights the importance of the non-diagonal entries in the covariance matrix.

### Comparison between DBNN and other popular methods

#### CB513 dataset

The best models developed in this work, DBNN, is then tested on the CB513 dataset and compared to other popular methods. Specifically, the methods SVM [8], PMSVM [11], SVMpsi [9], JNET [7], SPINE [6], and YASSPP [12] are selected for comparison, because they have been tested on the same (or a similar) dataset. Table 4 shows that DBNN has the best *Q*_3 _accuracy among all the methods mentioned above, with improvements ranging from 0.3% to 4.6%. Since the ErrSig is 0.41/0.40, this indicates that for all methods except YASSPP, the improvement made by DBNN is significant. In *SOV *measure, DBNN ranks second, below YASSPP but above SVMpsi. The comparison of the Matthews' coefficients between DBNN and YASSPP indicates that the two methods are complementary and may be combined to obtain further improvement in the prediction accuracy: DBNN has a better *C*_H _while YASSPP has a better *C*_C_.

**Table 4:** Comparative performance of DBNN against other popular methods tested on CB513.

Method	*Q*_3 _(%)	*SOV *(%)	*C*_H_	*C*_E_	*C*_C_
SVM	73.5	--	0.65	0.53	0.54
PMSVM	75.2	--	0.71	0.61	0.61
SVMpsi	76.6	73.5	0.68	0.60	0.56
JNET	76.9	--	--	--	--
YASSPP	77.8	75.1	0.58	0.64	0.71
^†^SPINE	76.8	--	--	--	--
DBNN/ErrSig	78.1/0.41	74.0/0.62	0.74/0.01	0.64/0.01	0.60/0.01
^†^DBNN/ErrSig	78.0/0.40	74.0/0.62	0.74/0.01	0.64/0.01	0.60/0.01

The description of DBNN can be found in Methods. Entries marked with "--" mean that the data could not be obtained from literatures. JNET has been trained and tested on the CB480 dataset (a reduced version of CB513), while all other methods have been trained and tested on the CB513 dataset. Methods marked with "†" have been evaluated using ten-fold cross-validation, while others have been evaluated using seven-fold cross-validation.

#### EVA dataset

DBNN is also compared to some live prediction servers by using the EVAc6 dataset and EVA website. The methods selected to compare are: Prospect [29], PROF_king [30], SAM-T99 [31], PSIPRED [3], PROFsec (unpublished), and PHDpsi [32], and their evaluation results on EVAc6 are obtained directly from the EVA website [33]. Because not all sequences are tested against all methods, the EVAc6 dataset is rearranged into five subsets, and the comparison is made between methods that are tested on the same subset (see Table 5).

**Table 5:** Comparative performance of DBNN and consensus methods against other leading methods tested on EVAc6.

Method	*Q*_3 _(%)	*SOV *(%)	*C*_H_	*C*_E_	*C*_C_
**Subset 1 (80 chains)**					
Prospect	71.1	68.7	0.59	0.69	0.49
DBNN/ErrSig	78.8/1.34	74.8/1.74	0.72/0.03	0.64/0.04	0.62/0.02
**Subset 2 (175 chains)**					
PROF_king	71.7	66.9	0.62	0.68	0.49
DBNN/ErrSig	77.3/0.86	71.9/1.27	0.71/0.02	0.64/0.03	0.57/0.02
**Subset 3 (179 chains)**					
SAM-T99	77.1	74.4	0.66	0.68	0.53
DBNN/ErrSig	77.3/0.86	71.9/1.28	0.71/0.02	0.64/0.02	0.57/0.02
**Subset 4 (212 chains)**					
PSIPRED	77.8	75.4	0.69	0.74	0.56
PROFsec	76.7	74.8	0.68	0.72	0.56
PHDpsi	75.0	70.9	0.66	0.69	0.53
DBNN/ErrSig	77.8/0.79	72.4/1.16	0.71/0.02	0.65/0.02	0.58/0.01
**Subset 5 (73 chains)**					
SAM-T99	76.3	72.9	0.71	0.64	0.56
PSIPRED	75.8	72.1	0.70	0.64	0.57
PROFsec	75.3	73.0	0.68	0.61	0.54
PHDpsi	73.3	69.2	0.66	0.56	0.52
PROF_king	70.7	64.9	0.63	0.57	0.50
DBNN/ErrSig	76.4/1.48	72.4/2.06	0.73/0.04	0.67/0.04	0.59/0.03
CM1/ErrSig	77.2/1.14	73.2/1.87	0.73/0.04	0.66/0.04	0.58/0.02
CM2/ErrSig	77.7/1.17	73.4/1.78	0.74/0.04	0.67/0.04	0.60/0.02
CM3/ErrSig	78.1/1.17	74.4/1.76	0.75/0.04	0.67/0.04	0.60/0.02

DBNN and the three consensus methods (CM1, CM2, and CM3) developed in this work are compared with other leading methods on five subsets of EVAc6; each comparison is carried out with maximum number of common sequences. The results of the six existing methods, Prospect, PROF_king, SAM-T99, PROFsec, PHDpsi, and PSIPRED, are obtained directly from the EVA website.

Table 5 shows that DBNN has generally a better *Q*_3 _than all other existing methods. In addition, the ErrSigs indicate that, for Prospect, PROF_king, and PHDpsi, the improvement made by DBNN is significant. In *SOV*, however, DBNN is modest: it is better than Prospect, PROF_king, and PHDpsi, but less well than SAM-T99, PROFsec, and PSIPRED, as shown in Table 5. Note that DBNN has the best *C*_H _among all the methods.

The *t*-tests are also performed for rigorous pairwise comparison between different methods. Specifically, we test the hypothesis that "method X" gives a significantly higher mean score than "method Y", by calculated *t*-values as $t=\bar{d}/\sigma \sqrt{n}$, where *d *= (*x*-*y*); *x *is the accuracy score of "method X", and *y *is of "method Y"; $\sigma =\sqrt{\left(\sum d^{2}-[{\left(\sum d\right)}^{2}/n]/(n-1\right)}$, and *n *= the number of proteins. We have evaluated all the methods on the subset 5 of EVAc6 (containing 73 chains), of which the prediction data of existing methods can be obtained directly from EVA website (Prospect is removed from the comparison because of the too many missing data for this method). The results shown in Table 6 indicate that DBNN has significantly better prediction, in both *Q*_3 _and *SOV*, than PROF_king and PHDpsi, and has competitive performance to the three state-of-the-arts: PSIPRED, SAM-T99, and PROFsec.

**Table 6:** Calculated *t*-values for differences in accuracy scores.

	Method Y								
	
Method X	PROF_king	SAM-T99	PSIPRED	PROFsec	PHDpsi	DBNN	CM1	CM2	CM3
***Q*_3_:**									
PROF_king	--	-4.70	-3.99	-3.56	-1.88	-4.52	-6.19	-6.93	-6.88
SAM_T99	4.70	--	0.50	0.93	2.45	-0.16	-1.41	-2.09	-3.02
PSIPRED	3.99	-0.50	--	0.53	2.18	-0.63	-2.01	-2.62	-3.38
PROFsec	3.56	-0.93	-0.53	--	2.31	-0.94	-2.87	-3.22	-3.72
PHDpsi	1.88	-2.45	-2.18	-2.31	--	-2.48	-4.55	-5.11	-5.10
DBNN	4.52	0.16	0.63	0.94	2.48	--	-0.91	-1.61	-2.50
CM1	6.19	1.41	2.01	2.87	4.55	0.91	--	-1.65	-2.82
CM2	6.93	2.09	2.62	3.22	5.11	1.61	1.65	--	-1.48
CM3	6.88	3.02	3.38	3.72	5.10	2.50	2.82	1.48	--
***SOV*:**									
PROF_king	--	-4.05	-3.89	-3.80	-1.99	-3.69	-5.30	-5.66	-5.86
SAM_T99	4.05	--	0.54	-0.06	2.43	0.36	-0.20	-0.35	-1.21
PSIPRED	3.89	-0.54	--	-0.62	1.77	-0.19	-0.97	-1.22	-2.57
PROFsec	3.80	0.06	0.62	--	2.93	0.37	-0.15	-0.28	-1.12
PHDpsi	1.99	-2.43	-1.77	-2.93	--	-1.67	-3.30	-3.30	-3.82
DBNN	3.69	-0.36	0.19	-0.37	1.67	--	-0.58	-0.83	-1.83
CM1	5.30	0.20	0.97	0.15	3.30	0.58	--	-0.27	-2.03
CM2	5.66	0.35	1.22	0.28	3.30	0.83	0.27	--	-2.55
CM3	5.86	1.21	2.57	1.12	3.82	1.83	2.03	2.55	--

The *t*-values are calculated for the differences in accuracy scores between "method X" and "method Y" (x-y) tested on EVAc6 subset 5. The descriptions of DBNN, CM1, CM2 and CM3 can be found in the text. Underlined are where calculated *t *> tabulated *t *(significant). The tabulated *t *= 1.67 for *α *= 0.05 and degree of freedom = 72.

All the above evaluation work shows that prediction accuracy of protein secondary structure by any individual program seems to reach a limit, no better *Q*_3 _than 78% (see Table 5). Previous studies [21,34] show that a simple way to achieve further improvement is to construct a consensus over several independent predictors. The consensus would be effective if the individual predictors are mutually complementary (more independent). So, the study of consensus performance is also a way to judge if a new method or program brings in new (complementary) information. This study is carried out with a design of three consensus methods (CM) using a simple "weighted vote" strategy to generate the final output: CM1 combines the five existing popular methods, PROF_king, SAM-T99, PSIPRED, PROFsec, and PHDpsi; CM2 repeatedly replaces one of the above five methods by DBN_final_, and CM3 is the same as CM2 except DBNN is in the place of DBN_final_. The weight for the vote of each method is set to be the success rate of the method for each type of secondary structure, which is derived from an individual evaluation of its own. The CM-series are evaluated on the subset 5 of EVAc6. The results shown in Table 5 indicate that CM3 has the top performance and that DBNN brings in complementary information to the family of existing methods. Note that CM2 ranks second (better than CM1 in both *Q*_3 _and *SOV*), indicating that the success of DBNN is derived from DBN.

The *t*-tests between the CM-series and the individual methods are also performed, and the results shown in Table 6 indicate that a simple combination of the five existing methods does not make significant improvement in accuracy: the individual method SAM-T99 has competitive *Q*_3 _to CM1. On the other hand, the inclusion of DBN or DBNN (both CM2 and CM3) has given rise to significantly better *Q*_3 _than all individual methods including SAM-T99. This is further enhanced by a direct comparison between CM3 and CM1; significant improvements in both *Q*_3 _and *SOV *are clearly evidenced. Finally, let us note that none of the consensus methods shows significant improvement in *SOV *over all individual methods, indicating that *SOV *is particularly hard to improve.

## Conclusion

A new method for protein secondary structure prediction of probabilistic nature based on dynamic Bayesian networks is developed and evaluated by several measures, which has shown significantly better prediction accuracy than previous pure HMM-type methods such as HMMCrooks and HMMChu. The improvement is mainly due to the use of a multivariate Gaussian distribution for the PSI-BLAST profile of each residue and the consideration of dependency between profiles of neighboring residues. In addition, because of the introduction of secondary structure segment length distributions in the model, DBN shows much better *SOV *than a typical NN.

The essentially different nature of DBN and NN inspires a model that combines the two and forms the DBNN with significant further improvements in both *Q*_3 _and *SOV*. DBNN is shown to be better than most of popular methods and competitive compared to the three state-of-the-art programs. We are then encouraged to explore further with consensus methods that combine all the best existing methods together. This study has demonstrated again the uniqueness of DBNN: the best consensus method is achieved by the inclusion of DBNN. This provides the evidence that DBNN brings in complementary information to the family of existing methods.

An interesting feature of our work here, compared to NN or SVM, is that it provides a set of distributions which have specific meanings and which can be studied further to improve our understanding of the model's behavior behind the prediction. An example is provided regarding the secondary structure segment length distributions used by the DBN, which is set to be an *a priori *distribution but can further be adjusted and improved. This points to a way for further improving the performance of DBN, by including modifications on more distributions, such as the transition probabilities between secondary structure states or the distribution of the profile of each residue. These distributions are also interesting for advancing the understanding of such fundamental problems as protein dynamics and protein folding, for which the information in implicit form in NN or SVM is of little use.

It appears that the limits of secondary structure prediction are being reached as no new method over the past decade has shown any major improvement since PSIPRED. All of the top methods are between 77%–80% accurate, in terms of *Q*_3_, depending on data set used. This implies that the complexity of the sequence-structure relationship is such that any single tool, when it attempts to extract (during learning) and to extrapolate (during predicting) the knowledge, can only represent some facets of this relationship, but not the whole. Further hope lies in the possibility that more facets are covered by new models, and that new models are integrated with the existing ones. The consensus methods reported above are just a simple approach in that direction; more sophisticated strategy for combining multiple scores can be sought in the future.

## Methods

### Generation of the PSI-BLAST profile

Each protein sequence in the datasets described above is used as query to search against the NR database [35] by using PSI-BLAST program [36]. The number of iterations in running PSI-BLAST is set to be 3; all other options are set to be defaults. The PSSM produced by the program is a matrix of integers typically in the range of ± 7 (see Fig. 2(a)). Each row of the PSSM is a 20-dimension vector corresponding to 20 amino acids, which is used to derive the PSI-BLAST profile of the corresponding residue.

### Transformation of the PSSM

Similar to other secondary structure prediction methods [3,6,11], we transform the PSSM into the range from 0 to 1 before using it as input of models. Two strategies are employed for the transformation: one follows the function

(3) $$f_{linear}(x)=\{\begin{array}{ll} 0, & \text{if }x<-7;\\ 1, & \text{if }x>7;\\ x/14+0.5, & \text{if }-7\leq x\leq 7. \end{array},$$

and is referred to as "linear transformation"; the other follows the function

(4) $$f_{sigmoid}(x)=\frac{1}{1+e^{-x}},$$

and is referred to as "sigmoid transformation".

### Assessment of the prediction accuracy

Several measures are adopted to assess the performance of our methods in a comprehensive way. The first is the overall three-state prediction accuracy, *Q*_3_, defined by

(5) $$Q_{3}=\frac{n}{N}\times 100,$$

where *n *is the number of correctly predicted residues and *N *is the total number of residues. The second, *SOV*, is a segment-level measure of the prediction accuracy, and its most recent definition can be found in [37]. At last, the Matthews' correlation coefficient [28] is used for each class of secondary structure, which is defined by

(6) $$C_{i}=\frac{n_{i}m_{i}-u_{i}o_{i}}{\sqrt{\left(n_{i}+u_{i})(n_{i}+o_{i})(m_{i}+u_{i})(m_{i}+o_{i}\right)}},$$

where *n*_*i *_is the number of residues correctly predicted to be secondary structure of class *i*, *m*_*i *_is the number of residues correctly not predicted to be secondary structure of class *i*, *u*_*i *_is the number of residues observed but not predicted to be secondary structure of class *i*, and *o*_*i *_is the number of residues predicted but not observed to be secondary structure of class *i *(*i *= H, E, and C).

### The dynamic Bayesian network

DBN is a directed graphical model in which nodes represent random variables and arcs represent dependency between nodes. The architecture of our DBN model is illustrated in Fig. 2(b). There are totally six nodes for each residue. Specifically, the node *AA*_*i *_(*i *= 1, 2, 3...) contains the PSI-BLAST profile of residue *i*, which is a 20-dimensional vector corresponding to 20 scores in the PSSM. The node *R*_*i *_stores replica of the profiles of a series of residues before *i*, i.e. the profiles of residues *i*-1, *i*-2, *i*-3, ... *i*-*L*_*AA*_, as shown in Fig. 2(b), where *L*_*AA *_is a profile window size indicating the range of the dependency for the profiles. As shown in Fig. 2(b), all the dependency between *AA*_*i *_and its neighboring sites, *AA*_*i*-1_, *AA*_*i*-2_, ... *AA*_*i*-*LAA*, _can be summarized into one single connection to *R*_*i*_, simplifying the topology of the graph. The state-space of *R*_*i *_is 21·*L*_*AA*_-dimensional, with 20·*L*_*AA *_storing the profiles of the past residues and extra *L*_*AA *_dimensions representing the "over-terminus" state.

The node *SS*_*i *_is used to describe the secondary structure state of residue *i*, which has a discrete state-space of three elements: H, E, and C. The node *d*_*i *_has a similar role as *R*_*i*_, but describes here the joint distribution with the secondary structure states of residues *i*-1, *i*-2, ... *i*-*L*_*SS*_, where *L*_*SS *_is the secondary structure window size indicating the range of the dependency, as shown in Fig. 2(b). Again, the node *d*_*i *_is introduced to simplify the topology of the graph, yet to keep a long-range dependency between profile (*AA*_*i*_) and secondary structure (*SS*_*i*-1_, *SS*_*i*-2_, ...). The dimension of *d*_*i *_is 4·*L*_*SS*_, where 3·*L*_*SS *_are from the joint past secondary structure states and the extra *L*_*SS *_from the "over-terminus" situation.

The nodes *D*_*i *_and *F*_*i *_are introduced to mimic a duration-HMM [22], with a specified parameter *D*_*max *_and two elements, respectively. Specifically, *D*_*i *_represents the distance (measured by the number of residues) from the position *i *to the end of the corresponding secondary structure segment. For example, in a segment with end residue at position *j*, the value of *D*_*i *_is set to be *j*-*i*+1. Note that the state-space of *D*_*i *_requires that the maximum length of segments should not exceed *D*_*max*_. In order to cope with longer segments, a modified definition of *D*_*i *_is introduced as following: when the length of a segment ≤ *D*_*max*_, the value of *D*_*i *_is set as described above; when the length of the segment > *D*_*max*_, for example *D*_*max*_+3, the *D*_*i *_is set to be *D*_*max *_for the first four residues of the segment and is set to be *D*_*max*_-1, *D*_*max*_-2, ... 1 for the rest. In this way, the lengths of segments longer than *D*_*max *_are modeled by a geometric distribution (see below). The value of the node *F*_*i *_is deterministically dependent on *D*_*i*_: if *D*_*i *_> 1, *F*_*i *_= 1; if *D*_*i *_= 1, *F*_*i *_= 2.

Each node described above is assigned a specific conditional probability distribution (CPD) function according to the connections' pattern shown in Fig. 2(b), except for *R*_*i*_, which is a "root" node [22] with no "parent node", and which is observable in both training and predicting. Specifically, the CPD of *AA*_*i *_(*i *= 1, 2, 3) is modeled using a conditional linear Gaussian function, which is defined by:

(7) *P*(*AA*_*i *_= **y **| *R*_*i *_= **u**, *SS*_*i *_= *α*, *d*_*i*_ = *γ*) = *N*(**y**;**w**_*α*,*γ*_**u **+ **c**_*α*,*γ*_, Σ_*α*,*γ*_),

where *N*(**y**;**μ**, **Σ**) represents a Gaussian distribution with mean **μ **and covariance **Σ**, **u **is a 21·*L*_*AA*_-dimensional vector, *α *is one of H, E, and C, and *γ *is one of the *L*_*SS*_-tuples formed by four elements: O, H, E, and C (O represents the "over-terminus" state). The distribution function is characterized by the mean **μ**_*α*,*γ *_= **w**_*α*,*γ*_**u **+ **c**_*α*,*γ*_, where **w**_*α*,*γ *_is a 20 × 21 *L*_*AA *_matrix and **c**_*α*,*γ *_is a 20-dimensional vector, and the covariance **Σ**_*α*,*γ*_. The subscripts *α *and *γ *indicate that the parameters **w**_*α*,*γ*_, **c**_*α*,*γ*_, and **Σ**_*α*,*γ *_are dependent on the states of *SS*_*i *_and *d*_*i*_. Second, the CPD of *SS*_*i *_(*i *= 2, 3, 4...) is defined by

(8) $$P(SS_{i}=\beta |SS_{i-1}=\alpha ,F_{i-1})=\{\begin{array}{ll} 1, & \text{if }\beta =\alpha \text{ and }F_{i-1}=1;\\ 0, & \text{if }\beta \neq \alpha \text{ and }F_{i-1}=1;\\ T_{\alpha }(\beta ), & \text{if }F_{i-1}=2. \end{array},$$

where *T*_*α*_(*β*) is the transition probability from the secondary structure state *α *to the state *β*. Third, the CPD of *d*_*i *_(*i *= 2, 3, 4...) is defined by

(9) $$P(d_{i}=\lambda |SS_{i-1}=\alpha ,d_{i-1}=\gamma )=\{\begin{array}{ll} 1 & \text{if }\lambda_{\text{1}}=\gamma_{2}\text{, }\lambda_{2}=\gamma_{3}\text{, }...\lambda_{Lss\text{-}1}=\gamma_{Lss}\text{,}\\ & \text{and }\lambda_{Lss}=\alpha ;\\ 0 & \text{otherwise}\text{.} \end{array},$$

where *λ*_*j *_and *γ*_*j *_(*j *= 1, 2, ... *L*_*SS*_) are the *j*th elements of the *L*_*SS*_-tuples *λ *and *γ*, respectively. Fourth, the CPD of *D*_*i *_(*i *= 2, 3, 4...) is defined by

(10) $$\begin{array}{l} P(D_{i}=n|D_{i-1}=m,SS_{i}=\alpha ,F_{i-1})\\ =\{\begin{array}{ll} h_{\alpha }, & \text{if }m=D_{\max}\text{, }n=m,\text{ and }F_{i-1}=1;\\ 1-h_{\alpha }, & \text{if }m=D_{\max},\;n=m-1,\text{ and }F_{i-1}=1;\\ 1, & \text{if }m<D_{\max}\text{, }n=m-1\text{, and }F_{i-1}=1;\\ 0, & \text{if }m\text{ and }n\text{ have other values, and }F_{i-1}=1;\;\\ g_{\alpha }\text{(}n\text{),} & \text{if }F_{i-1}=2. \end{array}, \end{array}$$

where *g*_*α*_(*n*) is the segment length distribution given the secondary structure state *α *and *h*_*α *_is the probability for *D*_*i *_to maintain the value *D*_*max *_given *SS*_*i *_= *α *and *D*_*i*-1 _= *D*_*max*_. Using this function, the probability of producing a segment with length *n *(*n *> = *D*_*max*_) is proportional to (1-*h*_*α*_)*h*_*α*_^*n*-*Dmax*^, i.e. a geometric distribution. The validity of using such a distribution to model segments of length longer than *D*_*max *_is supported by Fig. 3(a), in which all the helices, sheets, and coils show exponential tails in their segment length distributions. Fig. 3(a) also indicates that a proper *D*_*max *_should be 13, after which all the distributions can be fitted well to exponential functions (see the inset of Fig. 3(a)). At last, the CPD of *F*_*i *_(*i *= 1, 2, 3...) is defined by

(11) $$P(F_{i}|D_{i})=\{\begin{array}{ll} 1, & \text{if }D_{i}>1\text{ and }F_{i}=1\text{, or }D_{i}=1\text{ and }F_{i}=2;\\ 0,\; & \text{otherwise}\text{.} \end{array}.$$

Note that the CPDs of *SS*_1_, *d*_1_, and *D*_1 _have similar definition to CPDs of *SS*_*i*_, *d*_*i*_, and *D*_*i *_(*i *= 2, 3, 4...) but with an independent set of parameters.

The parameters of the CPDs described above are derived by applying the maximum likelihood (ML) method to the training set. In prediction, the marginal probability distribution of *SS*_*i *_(*i *= 1, 2, 3...) is computed by using the forward-backward (FB) algorithm [22], and then the state of *SS*_*i *_with the maximum probability is the prediction of residue *i*. Both ML and FB algorithms are implemented by using the Bayes Net Toolbox [38].

### The neural network

The typical three-layered feed-forward back-propagation architecture is used in our NN-based models. The sliding window-based training and testing strategy are employed with an optimal window size of 15 derived from an empirical evaluation of varying window sizes from 7 to 19. The momentum terms and learning rates of the network are set to be 0.9 and 0.005, respectively, and the number of hidden units is set to be 75.

### Training and combinations

Training is done in two different ways, depending on datasets involved. For the dataset CB513 and SD576, the standard *N*-fold cross-validation testing strategy is adopted, where *N *is either 7 or 10. That is, the dataset is split into *N *subsets with approximately equal numbers of sequences in each, and then *N*-1 of them are used for training while the remaining one is used for testing; the process continues *N *times with a rotation of the testing subset, making sure that every protein sequence is tested once. The second way of training concerns the dataset EVAc6, for which there exists a separate large dataset EVAtrain with low sequence identity (< 25%) to EVAc6. So, it is customary to use EVAtrain as the training set and EVAc6 as the test set.

Note that the DBN and NN models are usually trained on the same training set, in order to make a comparison and to be combined later to form DBNN. However, the detailed training process of DBN is somewhat different from NN, owing to different architectures of the model. The DBN takes two sets of data as input, one for profile and the other for secondary structure; each set is a sliding window with the "current" residue located at the right end. The correlation information between "current" residue and its neighbors is stored in the data, but depends on the direction in which the window slides (from N-terminus to C-terminus or reverse). We actually run the DBN model in both directions and then average the results (see below). On the other hand, the NN takes only one sliding-window, with the "current" residue located at the center of the window. Finally, the training for DBNN is simple the training of DBN and NN on the same dataset.

When a sequence is selected for either training or testing, the original PSSM generated by PSI-BLAST can be transformed into [0 1] in two strategies: linear transformation [Eq. (3)] or sigmoid transformation [Eq. (4)]. In addition, as mentioned above, the direction from either N-terminus to C-terminus (NC) or the reverse (CN) gives rise to different correlation structure, so we treat them separately. As a result, four basic DBN models are generated corresponding to four above combinations: (i) DBN_linear+NC_, (ii) DBN_linear+CN_, (iii) DBN_sigmoid+NC_, and (iv) DBN_sigmoid+CN_, where the subscripts are self-explanatory. On the other hand, NN is split into two kinds according to the transformation for PSSM, and the corresponding models are denoted by NN_linear _and NN_sigmoid_, respectively.

The six basic models described above are believed to contain complementary information and need to be combined to form three final models. Two strategies for forming the final models are used. The first is a simple averaging of the output scores and is used to form the two architecture-based final models, DBN_final _and NN_final_. It is done in two steps. One first averages the outputs of DBN_linear+NC _and DBN_linear+CN _to form DBN_linear_, and of DBN_sigmoid+NC _and DBN_sigmoid+CN _to form DBN_sigmoid_. Then, DBN_linear _and DBN_sigmoid _are further combined to form DBN_final_. Similarly, NN_linear _and NN_sigmoid _are combined to form NN_final_.

The second strategy consists in using a new neural network, which has the same architecture to basic NN models except that it takes as inputs, the outputs of all the other scores (DBN_linear+NC_, DBN_linear+CN_, DBN_sigmoid+NC_, DBN_sigmoid+CN_, NN_linear_, and NN_sigmoid_). This final model is named DBNN, and is the one that shows the best performance among the models mentioned above.

## Availability

All the codes and datasets described above are available from our homepage [39].

## Authors' contributions

ZSS and HQZ supervised the whole process of the work. XQY wrote the codes and did the tests. XQY, HQZ, and ZSS draft the manuscript.

## References

[B1] Qian N, Sejnowski TJ (1988). Predicting the secondary structure of globular proteins using neural network models. J Mol Biol.

[B2] Rost B, Sander C (1993). Prediction of protein secondary structure at better than 70% accuracy. J Mol Biol.

[B3] Jones DT (1999). Protein secondary structure prediction based on position-specific scoring matrices. J Mol Biol.

[B4] Pollastri G, McLysaght A (2005). Porter: a new, accurate server for protein secondary structure prediciton. Bioinformatics.

[B5] Adamczak R, Porollo A, Meller J (2005). Combining prediction of secondary structure and solvent accessiblility in proteins. Proteins.

[B6] Dor O, Zhou Y (2007). Achieving 80% ten-fold cross-validated accuracy for secondary structure prediction by large-scale training. Proteins.

[B7] Cuff JA, Barton GJ (2000). Application of multiple sequence alignment profiles to improve protein secondary structure prediction. Proteins.

[B8] Hua S, Sun Z (2001). A novel method of protein secondary structure prediction with high segment overlap measure: support vector machine approach. J Mol Biol.

[B9] Kim H, Park H (2003). Protein secondary structure prediction based on an improved support vector machines approach. Protein Eng.

[B10] Ward JJ, McGuffin LJ, Buxton BF, Jones DT (2003). Secondary structure prediction with support vector machines. Bioinformatics.

[B11] Guo J, Chen H, Sun Z, Lin Y (2004). A novel method for protein secondary structure prediction using dual-layer SVM and profiles. Proteins.

[B12] Karypis G (2006). YASSPP: better kernels and coding schemes lead to improvements in protein secondary structure prediction. Proteins.

[B13] Stultz CM, White JV, Smith TF (1993). Structural analysis based on state-space modeling. Protein Sci.

[B14] Thompson MJ, Goldstein RA (1997). Predicting protein secondary structure with probabilistic schemata of evolutionarily derived information. Protein Sci.

[B15] Schmidler SC, Liu JS, Brutlag DL (2000). Bayesian segmentation of protein secondary structure. J Comput Biol.

[B16] Crooks GE, Brenner SE (2004). Protein secondary structure: entropy, correlations and prediction. Bioinformatics.

[B17] Aydin Z, Altunbasak Y, Borodovsky M (2006). Protein secondary structure prediction for a single-sequence using hidden semi-Markov models. BMC Bioinformatics.

[B18] Chu W, Ghahramani Z, Podtelezhnikov A, Wild DL (2006). Bayesian segmental models with multiple sequence alignment profiles for protein secondary structure and contact map prediction. IEEE Trans Comput Biol Bioinfo.

[B19] Karplus K, Katzman S, Shackleford G, Koeva M, Draper J, Barnes B, Soriano M, Hughey R (2005). SAM-T04: what is new in protein-structure prediction for CASP6. Proteins.

[B20] Prediction scheme of SAM-T04. http://www.soe.ucsc.edu/research/compbio/SAM_T06/faq.html.

[B21] Cuff JA, Barton GJ (1999). Evaluation and improvement of multiple sequence methods for protein secondary structure prediction. Proteins.

[B22] Murphy KB (2002). Dynamic Bayesian networks: representation, inference and learning. Computer Science.

[B23] Koh IY, Eyrich VA, Marti-Renom MA, Przybylski D, Madhusudhan MS, Eswar N, Grana O, Pazos F, Valencia A, Sali A, Rost B (2003). EVA: evaluation of protein structure prediction servers. Nucleic Acids Res.

[B24] Berman HM, Westbrook J, Feng Z, Gilliland G, Bhat TN, Weissig H, Shindyalov IN, Bourne PE (2000). The protein data bank. Nucleic Acids Res.

[B25] EVA common set 6. http://cubic.bioc.columbia.edu/eva/sec/set_com6.html.

[B26] Andreeva A, Howorth D, Brenner SE, Hubbard TJP, Chothia C, Murzin AG (2004). SCOP database in 2004: refinements integrate structure and sequence family data. Nucleic Acids Res.

[B27] Kabsch W, Sander C (1983). Dictionary of protein secondary structure: pattern recognition of hydrogen-bonded and geometrical features. Biopolymers.

[B28] Matthews BW (1975). Comparison of the predicted and observed secondary structure of T4 phage lysozyme. Biochim Biophys Acta.

[B29] Xu Y, Xu D (2000). Protein threading using PROSPECT: design and evaluation. Proteins.

[B30] Ouali M, King RD (2000). Cascaded multiple classifiers for secondary structure prediction. Protein Sci.

[B31] Karplus K, Barrett C, Cline M, Diekhans M, Grate L, Hughey R (1999). Predicting protein structure using only sequence information. Proteins.

[B32] Przybylski D, Rost B (2002). Alignments grow, secondary structure prediction improves. Proteins.

[B33] EVA results. http://cubic.bioc.columbia.edu/eva/sec/common3.html.

[B34] McGuffin LJ, Jones DT (2003). Benchmarking secondary structure prediction for fold recognition. Proteins.

[B35] NR database. ftp://ftp.ncbi.nih.gov/blast/db.

[B36] Altschul SF, Madden TL, Schaffer AA, Zhang J, Zhang Z, Miller W, Lipman DJ (1997). Gapped BLAST and PSI-BLAST: a new generation of protein database search programs. Nucleic Acids Res.

[B37] Zemla A, Venclovas C, Fidelis K, Rost B (1999). A modified definition of sov, a segment-based measure for protein secondary structure prediction assessment. Proteins.

[B38] Bayes net toolbox. http://bnt.sourceforge.net.

[B39] DBNN homepage. http://ctb.pku.edu.cn/main/SheGroup/Software/DBNN.

